# Biological activities of nitidine, a potential anti-malarial lead compound

**DOI:** 10.1186/1475-2875-11-67

**Published:** 2012-03-09

**Authors:** Jérome Bouquet, Marion Rivaud, Séverine Chevalley, Eric Deharo, Valérie Jullian, Alexis Valentin

**Affiliations:** 1 Université de Toulouse; UPS; UMR 152 (Laboratoire Pharmadev), Faculté de Pharmacie, 35 Chemin des maraîchers, F-31062 Toulouse cedex 9, France; 2 Institut de Recherche pour le Développement (IRD); UMR 152 (Laboratoire Pharmadev), 118, rte de Narbonne, F-31062 Toulouse cedex 9, France

## Abstract

**Background:**

Nitidine is thought to be the main active ingredient in several traditional anti-malarial remedies used in different parts of the world. The widespread use of these therapies stresses the importance of studying this molecule in the context of malaria control. However, little is known about its potential as an anti-plasmodial drug, as well as its mechanism of action.

**Methods:**

In this study, the anti-malarial potential of nitidine was evaluated in vitro on CQ-sensitive and -resistant strains. The nitidine's selectivity index compared with cancerous and non-cancerous cell lines was then determined. In vivo assays were then performed, using the four-day Peter's test methodology. To gain information about nitidine's possible mode of action, its moment of action on the parasite cell cycle was studied, and its localization inside the parasite was determined using confocal microscopy. The in vitro abilities of nitidine to bind haem and to inhibit β-haematin formation were also demonstrated.

**Results:**

Nitidine showed similar in vitro activity in CQ-sensitive and resistant strains, and also a satisfying selectivity index (> 10) when compared with a non-cancerous cells line. Its in vivo activity was moderate; however, no sign of acute toxicity was observed during treatment. Nitidine's moment of action on the parasite cycle showed that it could not interfere with DNA replication; this was consistent with the observation that nitidine did not localize in the nucleus, but rather in the cytoplasm of the parasite. Nitidine was able to form a 1-1 complex with haem in vitro and also inhibited β-haematin formation with the same potency as chloroquine.

**Conclusion:**

Nitidine can be considered a potential anti-malarial lead compound. Its ability to complex haem and inhibit β-haematin formation suggests a mechanism of action similar to that of chloroquine. The anti-malarial activity of nitidine could therefore be improved by structural modification of this molecule to increase its penetration of the digestive vacuole in the parasite, where haemoglobin metabolization takes place.

## Background

Malaria is a major cause of childhood mortality and adult morbidity in many parts of the world. Recent estimates indicate that more than 200 million clinical episodes of malaria and approximately about 1 million deaths due to *Plasmodium falciparum *occur worldwide annually. Resistance of this parasite to virtually all of the currently available anti-malarial drugs is of great concern; consequently, new, inexpensive drugs are urgently needed to address the global burden of malaria. Natural substances have provided the best anti-malarials that are currently available. These anti-malarials, including artemisinin and quinine, as well as numerous molecules derived from plants, are promising lead compounds that combat malaria infection. Unfortunately, the efficacy of many of these molecules has only been confirmed by in vitro experiments on *P. falciparum *[1]. This is the case for nitidine, an alkaloid found in several traditional remedies from diverse endemic areas that had previously been discovered and has been rediscovered in the last 50 years.

Nitidine was first isolated in 1959 from *Zanthoxylum nitidium *(Rutaceae) [2]; it was found again approximately 40 years later in a traditional Kenyan anti-malarial remedy [3] and was more recently discovered in *Zanthoxylum rhoifolium *(Rutaceae), a traditional remedy from South America [4]. The widespread use of nitidine stresses the importance of this molecule in the field of malaria control.

Many biological properties have been ascribed to nitidine, including its use as an anti-microbial [5], anti-HIV [6] analgesic and anti-inflammatory [7] agent. Cytotoxic and anti-cancerous properties were also reported [8], including nitidine-mediated inhibition of topoisomerase I [9], which may indicate potentially important toxicity and could rule out this molecule's use as an anti-malarial drug.

Further investigations concerning the anti-malarial activity of nitidine, with focus on its toxicity on different cells lines, its in vitro activity on chloroquine-resistant *P. falciparum *strains, and its in vivo activity in a murine malaria model were pursued. To obtain insight into nitidine's mechanism of action, its interaction with haemin and its ability to inhibit β-haematin formation were studied.

## Methods

### Chemicals

**Figure 1:**
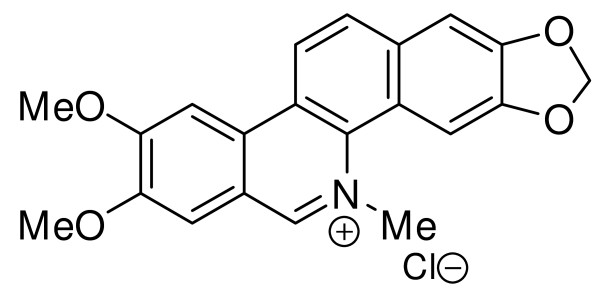
**Nitidine chloride**.

All of the chemicals used in this study were obtained from Sigma-Aldrich (Saint-Quentin, France). Nitidine in its basic form was purified from *Z. rhoifolium *as previously described [4]. Basic nitidine was then dissolved in chloroform with 10% methanol, and the addition of concentrated hydrochloric acid at 0 C precipitated nitidine as a chloride salt. The precipitate was filtered, rinsed with water and dried under a vacuum. Its structure (Figure 1) was confirmed by 1H-NMR and 13 C-NMR mass spectrometry. Nitidine chloride appeared as a pure compound on the ^1^H-NMR spectra, therefore its purity can be considered superior to 95%. For biological uses, nitidine chloride was dissolved as a stock solution (10 mg/ml) in DMSO.

### In vitro anti-plasmodial activity

The *P. falciparum *F-32-Tanzania chloroquine-sensitive strain, FcM29 and FcB1-Columbia chloroquine-resistant strains were cultured according to Trager and Jensen [10], with modifications [11]. The cultures were synchronized by a combination of magnetic concentration and 5% D-sorbitol lysis (Merck, Darmstadt, Germany) [12,13]. The F-32-Tanzania strain was considered to be chloroquine-sensitive (chloroquine IC50: 38 ± 6 nM); the FcM29 and FcB1-Columbia strains were considered to be chloroquine-resistant (chloroquine IC50: 170 ± 32 nM and 230 ± 11 nM, 196 ± 31 nM, and > 100 nM, respectively). Anti-plasmodial activity was determined by the [^3^H]-hypoxanthine (Amersham-France) incorporation method [14]. The resistance index was calculated as follows: IC_50 _F32/IC_50 _FcB1 and IC_50 _F32/IC_50 _FcM29

The sensitivity of different stages of *P. falciparum *was tested using the FcB1 strain. Serial dilutions of nitidine, which were close to its IC_50 _determined previously on this strain, were prepared. After synchronization over a six-hour period (time between magnetic collection of previous stages and sorbitol lysis after invasion), the parasites were plated at ring stage in 24-well plates. The drugs (nitidine and chloroquine as a control) were added, and the plates were incubated for 8 h; the corresponding wells were then washed while the drugs were added into the new wells for another eight-hour incubation. The cultures were then incubated until the end of the erythrocytic cycle plus an additional 24 h. Giemsa-stained thin smears were made, and parasitaemia was confirmed by the numeration of at least 10,000 erythrocytes [15].

### In vitro cytotoxicity

The toxicity of nitidine was estimated using MCF-7 cells (human breast carcinoma) and Vero cells (normal monkey kidney cells). These cell lines were cultured in the same conditions as *P. falciparum*, except for the replacement of 5% human serum with 10% foetal calf serum. After the addition of nitidine at increasing concentrations, cell growth was estimated by [^3^H]-hypoxanthine incorporation following a 48-h incubation and was compared with a control sample that did not have additional chemicals (the mean of the corresponding wells was referred to as 100%) [15]. The selectivity index was calculated as follows: IC_50 _FcB1/IC_50 _McF7 and IC_50 _FcB1/IC_50 _Vero.

### In vivo anti-malarial activity testing

The classic four-day suppressive in vivo assay was performed using CD female mice according to European legislation on laboratory animal use and care (EEC directive 86/609). The mice (mean body weight: 20 ± 2 g) were infected with 10^6 ^*Plasmodium vinckei petteri*-infected red blood cells [16] in RPMI on day 0. Groups of five mice were treated intra-peritonally from days 0-3 with increasing doses (0.1 mg/kg to 20 mg/kg) of the drug. On day 4, Giemsa-stained smears were made for each mouse, and parasitaemia was estimated by visual numeration of at least 5,000 erythrocytes. Mice treated with RPMI alone served as negative controls, and mice treated with chloroquine at various doses served as positive controls. Inhibition percentage was calculated using the following formula: (control parasitaemia - parasitaemia with drugs)/(control parasitaemia) × 100.

### Interaction with haem

The binding of nitidine with haemin chloride was assessed using UV-Visible spectroscopy (Analitik Jena Specord 205). Haemin chloride (Sigma 51280) was used without further purification. The H_2_O used during the UV studies was freshly filtered milli-q water. To minimize the aggregation of haemin on surfaces, only carefully washed glass and quartz were used (glass volumetric flasks, glass pipettes, and microsyringes, quartz UV cuve), as previously described [17]. The UV interaction experiments were conducted in a 40/60 DMSO/H_2_O mixture buffered with HEPES, as described by Egan et al. [18], with modifications. Haemin solution (2 μM, sol C) was titrated by the addition of increasing amounts of a solution of nitidine mixed with haemin (sol D) to ensure a constant haemin concentration during titration. The solutions were prepared in volumetric flasks. Mother solutions of haemin (1 mM, sol A) and nitidine (1 mM, sol B) in DMSO were freshly prepared. To obtain the haemin solution (sol C), sol A (20 μl) was mixed with DMSO (4 ml) and 0.2 M HEPES Buffer (0.5 ml), and the volume was adjusted to 10 ml with water. To obtain the titration solution (sol D), sol A (10 μl) and sol B (1 ml) were mixed with DMSO (2 ml) and 0.2 M HEPES Buffer (0.5 ml), and the volume was adjusted to 5 ml with water.

Two ml of haemin solution (sol C) were poured into a quartz UV cuvette, and increasing amounts of titrant (sol D) were added (5 μl was added 42 times, 10 μl was added ten times, and 50 μl was added ten times).

Absorbance at 402 nm was recorded after each addition of titrant solution (sol D) and agitation of the cuvette content. As nitidine absorbed at 402 nm, a blank titration of nitidine under the same conditions as described above was prepared, but without haemin. The variation in absorbance (ΔA) for each point of the titration curve corresponded to the value obtained during the titration (A1) minus the value obtained during the blank experiment (A2), minus the value of haemin solution when no ligand was added (A = A1 - A2 - A(0)).

This binding experiment was also performed using chloroquine diphosphate (in water) as a positive control. All of the titration experiments were performed in triplicate.

Stoicheiometry and binding constants of the haem-nitine and haem-chloroquine complexes were calculated from titration curves using the SPECFIT32^® ^software.

### Inhibition of β-haematin formation

The protocol described by Deharo et al. [19] was followed. Briefly, in a normal, non-sterile flat-bottom 96-well plate, 50 μl of 0.5 mg/ml haemin chloride (Sigma 51280) freshly dissolved in DMSO was added to 100 μl acetate buffer (c = 0.2 M, pH = 4.4) and to 50 μl of a solution of nitidine chloride dissolved in DMSO (c = 1; 0.5; 0.1; 0.05; 0.01 mg/ml). The plate was agitated and left at 37 C for 18 h. Then the plate was centrifuged at 550 g for 8 min. The supernatant was removed by vigorously flipping the plate upside down. Then 200 μl DMSO was added to wash the pellet, the plate was agitated and centrifuged at 550 g for 8 min, and the supernatant was removed again. This washing procedure was repeated twice. Then the pellet was dissolved in 200 μl 0.1 M sodium hydroxide solution, and the absorbance was read at 405 nM. The results were expressed as per cent of inhibition compared to a blank experiment (DMSO). Each concentration was tested in triplicate. The positive control was quinine hydrochloride in DMSO. Each experiment was performed three times.

### Microscopy

The fluorescent properties of nitidine (excitation λ: 366 and 380 nm, emission peaks 440 (blue) and 540 (green) nm) were used to determine its localization and concentration in red blood cells. Approximately 100 μl of each culture was washed three times in complete culture medium, and nitidine was added at increasing concentrations (52 to 1040 μM, where the optimum was 120 μM). After a two-hour incubation, three washes were performed with complete culture medium, and then different dyes were added (Lysotraquer^® ^red (5 μM) and Draq5^® ^(5 μM)). Following an additional 30-min incubation, three washes using PBS were performed, and the culture was mounted for microscopic examination onto glass slides. For microphotography, glass slides were coated with poly-L-lysine (Sigma) prior to deposition of the culture. The slides were analysed with a Nikon (Eclipse E 400) microscope with DAPI and FITC filters. For confocal microscopy, a Leica sp2 AOBS device with a 405-nm laser diode and a Helium/Neon laser (543 nm ray and 633 nm ray) was used. None of the pictures were altered using the computer, with the exception of a mathematical superposition that was performed using Paint Shop Pro^® ^software.

## Results

The anti-plasmodial activity of nitidine was evaluated on three strains of *P. falciparum *with different sensitivities to chloroquine (Table 1). The values of IC_50 _for nitidine ranged from 0.49 to 0.80 μM. The resistance index (RI) of nitidine was lower than the RI of chloroquine (1.55 and 0.95 for F32/FcB1 and F32/FcM29, respectively, versus more than 4 for chloroquine, Table 1).

**Table 1:** *In vitro *activity (IC_50 _in μM) of tested drugs on *Plasmodium falciparum *in MCF7 and Vero cells

Cell lines	Nitidine	Chloroquine	Doxorubicin
F32	0.52 ± 0.1	0.051	NT

FcB1	0.80 ± 0.28	0.141	NT

FcM29	0.49 ± 0.1	0.22	NT

RI^a^	1.55 and 0.95	4.3	

MCF-7	0.23 ± 0.03	> 50	0.42 ± 0.05

Vero	8.16 ± 1.04	> 50	6.4 ± 0.9

SI^b^	0.74 and 26.3		

a: Resistance index: F32/FcB1 and F32/FcM29

b: Selectivity index: FcB1/McF7 and FcB1/Vero

**Figure 2:**
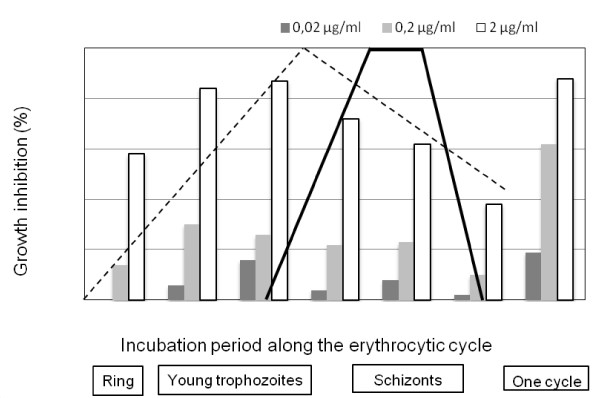
**Nitidine's moment of action on the erythrocytic cycle of *Plasmodium falciparum***. A parasite culture synchronized over a six-hour period was subjected to an eight-hour pulse of nitidine at IC_50 _(grey bars), 1/10 (dark grey) and 10 times the value (white bars). Major events along the erythrocytic cycle are summarized. The dotted line shows protein synthesis, and the solid black line shows DNA synthesis [20].

In an attempt to determine nitidine's moment of action during the erythrocytic cycle, various concentrations of nitidine were tested on a highly synchronized culture of *P. falciparum *(FcB1 strain). At concentrations ranging from one tenth to ten times the IC_50_, the highest inhibition of *P. falciparum *growth was obtained during the two first thirds of the cycle, with a maximum inhibition between hours 8 and 24 (Figure 2). Throughout the same experiments, when nitidine was added throughout the cycle, the observed values were similar to those obtained by [^3^H]-hypoxanthine incorporation (IC_50 _close to 0.52 μM).

Nitidine's cytotoxicity was then determined using two cell lines, MCF-7 and Vero. The IC_50 _for MCF-7 was close to the IC_50 _observed for *P. falciparum *and was much higher for Vero cells (SI 0.74 and 26.3 respectively).

Because Vero cells are normal (non-transformed cells), the toxicity observed on this cell line was low enough to perform an in vivo assay (Table 2). In the classic four-day in vivo suppressive test, parasitaemia decreased enough to determine the ED_50 _of nitidine using only two doses (18.9 mg/kg/day over four days). The positive control, chloroquine, displayed an ED_50 _value of 5 mg/kg/day over four days.

**Table 2:** *In vivo *activity of nitidine in a four-day suppressive test (ED_50_)

	n	Mean parasitaemia ± SD	inhibition
Control	10	69.8 ± 12.8	

CQ 10 mg/kg/d	5	1.1 ± 1.2	98%

CQ 1 mg/kg/d	5	78.9 ± 8.2	0%

Nitidine 10 mg/kg/d	5	62.1 ± 8.9	11%

Nitidine 20 mg/kg/d	5	28.5 ± 10.4	59%

In the haem-binding experiment, a good fit of the experimental titration curves with the calculated curves was obtained for nitidine and chloroquine when studying the formation of a 1-1 haem-molecule complex. This finding was in agreement with the literature [18]. With this model, a binding log K value of 5.17 ± 0.02 was obtained for chloroquine and 4.4 ± 0.3 for nitidine. The log K value for chloroquine in the literature is 5.5 [18]. However it can be noticed that a model incorporating the formation of both 1-1 and 2-1 haem-molecule complexes slightly improved the fit for chloroquine and also provided a good fit for nitidine.

In the in vitro inhibition of β-haematin formation assay, an IC_50 _of 18 ± 7 μM for nitidine was found, which was better than the value obtained for the quinine-positive control (78 ± 20 μM) and was in the same range as the value published for chloroquine (28 ± 5 μM) [19].

**Figure 3:**
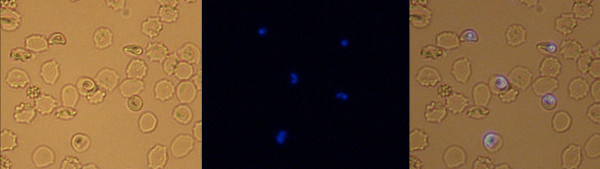
**Nitidine concentration in parasitized red blood cells (optical microscopy)**. The drug was added at a final concentration of 120 μM. a: direct examination (bar: 10 μm), b: FITC filter, c: merge. The drug appeared to be concentrated in the parasite inside the parasitized RBC.

**Figure 4:**
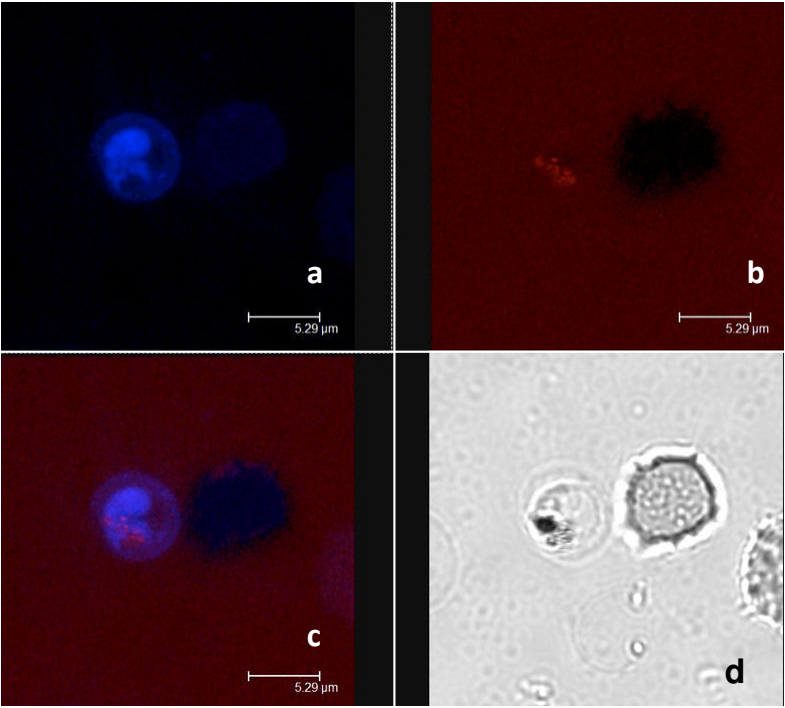
**Confocal microscopy analysis after double staining cells with nitidine and lysotracker^®^. a**: staining with 120 μM nitidine, **b**: staining with 5 μM lysotracker^®^, **c**: merge, **d**: daylight. The repartition of nitidine was not similar to that of Lysotracker^®^, a stain that concentrates in the food vacuole of the parasite.

**Figure 5:**
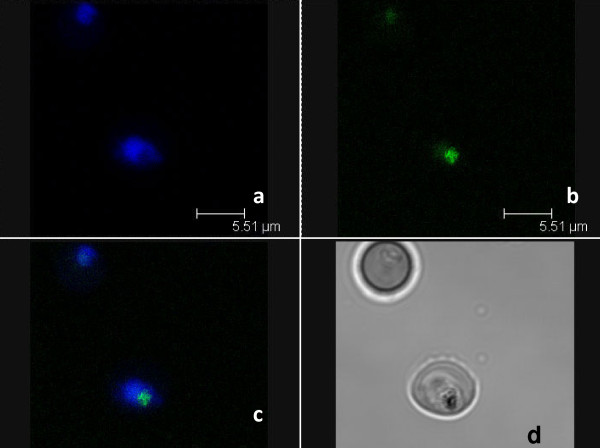
**Confocal microscopy after double staining cells with nitidine and Draq5^®^**. **a**: staining with 120 μM nitidine, **b**: staining with 5 μM Draq5^®^, **c**: merge, **d**: daylight. The repartition of nitidine was not similar to that of Draq^®^, a stain that concentrates in the nucleus of the parasite.

Microscopic analysis demonstrated that nitidine preferentially concentrated in parasitized red blood cells (Figure 3) independent of parasite stage. In an attempt to determine the precise intra-parasite localization, co-staining followed by confocal microscopy analysis was performed. After staining with nitidine, a double staining with digestive vacuole specific Lysotracker^® ^and nucleus specific draq5^® ^was performed (Figure 4 and 5). The resulting images demonstrated that nitidine did not settle in these two compartments. The fluorescence (corresponding to nitidine) was present in the whole parasite cytoplasm rather than in a restricted compartment. A range of nitidine concentrations was prepared in order to link fluorescence intensity (measured by confocal microscopy) to nitidine concentration. When the initial concentration of nitidine in the culture medium was 120 μM, the fluorescence intensity in the parasite was 5.5 mM.

## Discussion

Nitidine and fagaronine, another benzo[c]phenanthridine, have been described as the principle active anti-malarial compounds present in several traditional remedies and plants from different parts of the world [3,4,21]. Only one study focuses on the synthesis of anti-malarial benzo[c]phenanthridines [22]. In that context, studying the anti-malarial potency of nitidine was of potential interest. First, the in vitro anti-plasmodial activity of nitidine was independent of the chloroquine-resistance status of the *Plasmodium *strain (Table 1). The F32 strain has wild-type PfCRT, whereas FcB1 and FcM29 carry the K76T mutation; the latter strain carries numerous mutations that enhance CQ-resistance in PfMDR [23]. This means that nitidine is insensitive to the mechanism of chloroquine expulsion from chloroquine-resistant *Plasmodium *strains.

Because this molecule has been described as a potential anti-cancer agent, it was intersesting to assess its anti-plasmodial selectivity. Compared to MCF-7 cells, which derive from a cancerous cell line, no anti-plasmodial selectivity was observed; however, when compared to Vero cells, which derive from a non-cancerous mammalian cell line, the selectivity index was almost 30 times higher (SI = 26).

This finding highlights nitidine's ability to interfere with cell growth processes through a mechanism other than topoisomerase I inhibition [5]. This also seems to correspond with the low level of plasmodial topoisomerase I activity that was observed in the youngest stages of intraerythrocytic development of the parasite, which was in contrast to the high nitidine activity observed at the same stage [24].

This good selectivity index justified an in vivo experiment. In the four-day Peter's suppressive test, nitidine displayed an ED_50 _of 18.9/mg/kg/day over four days. This value was higher than that obtained for the chloroquine control (ED_50 _of 5/mg/kg/day over four days); however, no mice died during treatment, so the acute DL_50 _was not reached at 20 mg/kg/day, which was the highest dose. Thus, the low toxicity observed with Vero cells was confirmed.

The action of nitidine was highest during the first two thirds of the *P. falciparum *erythrocytic cycle (between hours 8 and 24), when the production of plasmodial RNA and protein were highest [20]. Therefore, nitidine did not interfere directly with plasmodial DNA synthesis, which occurs between hours 32 and 40 [20]. As anti-cancer activities of nitidine have been correlated with the ability of this molecule to intercalate into DNA [25] and to inhibit topoisomerase I [9], it can be hypothesized that nitidine could have a different and more specific anti-plasmodial mode of action. It can also be thaught that with its planar and aromatic structure, nitidine could interact with haem and exert its anti-plasmodial activity by inhibiting haemozoin formation inside the food vacuole of the parasite. This is now a widely noted mode of action for chloroquine, and promising new anti-malarial molecules have been developed based on a similar mechanism of action [26,27]. In vitro measurement of haem's interaction with anti-plasmodial molecules was described by Egan et al. [18]. Using a similar methodology, it was demonstrated that nitidine was able to form 1-1 complexes with haem, similar to chloroquine. The log K value obtained for these complexes showed that the nitidine-haem complex was less stable than the chloroquine haem-complex, but the affinity of nitidine for haem was higher than the affinities of quinine or mefloquine for haem, which were also reported by Egan.

Furthermore, it was evidenced that nitidine was able to inhibit β-haematin formation with the same potency as chloroquine in an in vitro test.

However, the localization experiment using confocal microscopy showed that nitidine was concentrated in the whole cytoplasm of *P. falciparum *and was not localized in the digestive vacuole where crystallization of haemozoin takes place. This fluorescence repartition may be due to the absence of nitidine in the digestive vacuole, or to the quenching of fluorescence when nitidine is complexed with haem, as previously reported for some cryptolepine derivatives [28]. Lastly, according to Ginsburg et al. [29] and Platel et al. [30], haem would be partially degraded in the plasmodial cytosol by GSH (reduced glutathione). Egan et al. [31] questioned this hypothesis but could not exclude the possibility that a small fraction of haem could be degraded in this location, thus providing the parasite's iron requirement. Consequently, it could be suggested that nitidine impairs the interaction between GSH and haem in the cytoplasm of the parasite instead of by direct action on the biocrystallization process in the vacuole.

Finally, nitidine was not localized in the nucleus and therefore does not interact with parasite DNA.

Structural modifications of nitidine could be used to improve its capacity to accumulate inside the food vacuole. It is well known that chloroquine accumulates inside the acidic food vacuole because of its basic amino side chain. Therefore, the synthesis of benzo[c]phenanthridine-bearing amino side-chains is currently underway. This strategy has been successfully employed in the xanthone and acridone series [27,32,33] and also in the cryptolepine and neocryptolepine series [28,34].

## Conclusion

This detailed study of the anti-plasmodial activity of nitidine showed that this known anti-plasmodial molecule, which is present in several traditional anti-malarial remedies, can be considered an interesting lead compound. Nitidine was active in vitro on chloroquine-sensitive and -resistant *Plasmodium *strains and displayed an adequate selectivity index when tested on a non-cancerous mammalian cell line. In vivo evaluation of nitidine showed that it had moderate anti-malarial activity and displayed no sign of acute toxicity. Its ability to bind haem and inhibit β-haematin formation in vitro suggests a possible chloroquine-like mechanism of action and paves the way for further structural optimization.

## Competing interests

The authors declare that they have no competing interests.

## Authors' contributions

JB performed the biological analysis, MR carried out the purification, chemical analysis and haem-nitidine interaction experiments. SC gave scientific assistance for parasite culture and synchronization and images in microscopy. ED participated to the elaboration of the manuscript. VJ conceived of the study, participated to its design and to the elaboration of the manuscript. AV conceived of the study, participated to its design and to the elaboration of the manuscript. All authors read and approved the final manuscript.
